# Meditation induces shifts in neural oscillations, brain complexity, and critical dynamics: novel insights from MEG

**DOI:** 10.1093/nc/niaf047

**Published:** 2025-11-23

**Authors:** Annalisa Pascarella, Philipp Thölke, David Meunier, Jordan O’Byrne, Tarek Lajnef, Antonino Raffone, Roberto Guidotti, Vittorio Pizzella, Laura Marzetti, Karim Jerbi

**Affiliations:** Institute for Applied Mathematics “M. Picone”, National Research Council, Via dei Taurini 19, 00185 Rome, Lazio, Italy; Department of Psychology, University of Montreal, Marie-Victorin Building, 90 avenue Vincent d'Indy, local D-418, Montreal, Quebec, Canada; Aix Marseille University, CNRS, INT, Institut de Neurosciences de la Timone, 27 Boulevard Jean Moulin, 13005 Marseille, France; Department of Psychology, University of Montreal, Marie-Victorin Building, 90 avenue Vincent d'Indy, local D-418, Montreal, Quebec, Canada; Department of Psychology, University of Montreal, Marie-Victorin Building, 90 avenue Vincent d'Indy, local D-418, Montreal, Quebec, Canada; Department of Psychology, Sapienza University of Rome, Via dei Marsi 78, 00185 Rome, Lazio, Italy; Department of Neuroscience, Imaging and Clinical Sciences, University G. D'Annunzio of Chieti-Pescara, Via dei Vestini 31, 66100 Chieti, Abruzzo, Italy; Institute for Advanced Biomedical Technologies, University G. D'Annunzio of Chieti-Pescara, Via Luigi Polacchi 11, 66100 Chieti, Abruzzo, Italy; Department of Neuroscience, Imaging and Clinical Sciences, University G. D'Annunzio of Chieti-Pescara, Via dei Vestini 31, 66100 Chieti, Abruzzo, Italy; Institute for Advanced Biomedical Technologies, University G. D'Annunzio of Chieti-Pescara, Via Luigi Polacchi 11, 66100 Chieti, Abruzzo, Italy; Institute for Advanced Biomedical Technologies, University G. D'Annunzio of Chieti-Pescara, Via Luigi Polacchi 11, 66100 Chieti, Abruzzo, Italy; Department of Engineering and Geology, University G. D'Annunzio of Chieti-Pescara, Viale Pindaro 42, 65127 Pescara, Abruzzo, Italy; Department of Psychology, University of Montreal, Marie-Victorin Building, 90 avenue Vincent d'Indy, local D-418, Montreal, Quebec, Canada; Mila, Quebec AI institute, 6666 Rue Saint-Urbain, Montreal, Quebec, Canada; UNIQUE Center, Quebec Neuro-AI Research Center, 3744 Jean-Brillant, Montreal, Quebec, Canada

**Keywords:** meditation, focused-attention meditation (FAM), open-monitoring meditation (OMM), complexity, criticality, magnetoencephalography

## Abstract

While the beneficial impacts of meditation are increasingly acknowledged, its underlying neural mechanisms remain poorly understood. We examined the electrophysiological brain signals of expert Buddhist monks during two established meditation methods known as Samatha and Vipassana, which employ focused attention and open-monitoring technique. By combining source-space magnetoencephalography with advanced signal processing and machine learning tools, we provide an unprecedented assessment of the role of brain oscillations, complexity, and criticality in meditation. In addition to power spectral density, we computed long-range temporal correlations (LRTC), deviation from criticality coefficient (DCC), Lempel–Ziv complexity, 1/f slope, Higuchi fractal dimension, and spectral entropy. Our findings indicate increased levels of neural signal complexity during both meditation practices compared to the resting state, alongside widespread reductions in gamma-band LRTC and 1/f slope. Importantly, the DCC analysis revealed a separation between Samatha and Vipassana, suggesting that their distinct phenomenological properties are mediated by specific computational characteristics of their dynamic states. Furthermore, in contrast to most previous reports, we observed a decrease in oscillatory gamma power during meditation, a divergence likely due to the correction of the power spectrum by the 1/f slope, which could reduce potential confounds from broadband 1/f activity. We discuss how these results advance our comprehension of the neural processes associated with focused attention and open-monitoring meditation practices.

## Introduction

Long relegated to the esoteric in the Western world, the practice and scientific study of meditation have seen a sharp rise in recent years. Meditation refers to a broad variety of practices and may be conceptualized as a family of complex emotional and attentional strategies developed for various ends, including the cultivation of well-being (Lutz et al. 2015). Over the past decades, a growing body of research has broadly supported the claim that meditation exerts beneficial effects on physical and mental health. Neuroimaging studies have begun to uncover the specific brain areas, networks, and underlying attentional processes that mediate its positive effects (Lutz et al. 2008; Raffone and Srinivasan 2010; Tang et al. 2015; Cooper et al. 2022). Expert meditators, in particular, offer a unique window into the neural dynamics associated with attention mechanisms (Marzetti et al. 2014; Guidotti et al. 2023) and altered states of consciousness.

Emerging theories suggest that many altered states of consciousness are quantifiable through measures of criticality and complexity, which index the brain’s information processing capacity (Schartner et al. 2015; Lendner et al. 2020; Varley et al. 2020; Zimmern 2020; Sarasso et al. 2021; Maschke et al. 2024). Criticality is the dynamical state of a system that operates at the boundary of a phase transition between an ordered phase and a disordered phase. The critical brain hypothesis proposes that the global neuronal dynamics of the healthy brain operate at this boundary or critical point, where emergent long-range correlations endow brain dynamics with both stability and flexibility, affording broadly optimal cognitive functioning (O’Byrne and Jerbi 2022; Hengen and Shew 2024). With regard to meditation and attentional processes, the brain’s ability to shift into focused attention has been linked to critical dynamics. Namely, according to a study by Irrmischer et al. (2018b), when the brain enters a focused task state, it appears to shift its dynamics away from criticality and into the ordered phase. The ordered phase is believed to facilitate exploitation by narrowing the range of possible responses, enhancing response stability, and minimizing distractions (Irrmischer et al. 2018b). The cited study, like many criticality studies in humans, examines critical dynamics at the level of neural oscillations (Hardstone et al. 2012). While the relationship between meditation and neural oscillations has been studied extensively (Le Van Quyen et al. 2001; Lomas et al. 2015; Merlet et al. 2024), research on the relation between meditation and critical brain dynamics, both in neuronal oscillations and beyond, is still at an early stage, with findings that remain unclear (Kakumanu et al. 2018; Irrmischer et al. 2018a; Dürschmid et al. 2020; Walter and Hinterberger 2022; D’Andrea et al. 2024).

The concept of complexity has been variously defined, but in general it is associated with measures of entropy and fractality. A recent review by Atad et al. (2025) highlighted a discrepancy in the literature on meditation and complexity, with empirical studies based on different theoretical frameworks suggesting that meditation is associated either with enhancement (Kakumanu et al. 2018; Lu and Rodriguez-Larios 2022; Walter and Hinterberger 2022) or reduction of brain signal complexity (Vyšata et al. 2014; Young et al. 2021). The clearest trends in the literature relate to expert meditators, namely that they show both (i) higher brain signal complexity (Kakumanu et al. 2018; Lu and Rodriguez-Larios 2022; Walter and Hinterberger 2022; D’Andrea et al. 2024) and (ii) attenuated long-range temporal correlations during meditation compared to waking rest or mind wandering (Irrmischer et al. 2018a; Walter and Hinterberger 2022; D’Andrea et al. 2024), as well as (iii) a reduced baseline (trait) complexity compared to novices and controls (Kakumanu et al. 2018). The authors also analyzed these results based on the various meditation styles, but found no differences. They suggest two possible interpretations: either complexity-related measures can characterize meditation regardless of technique, or the categorization used is too simplistic and does not accurately reflect meditators’ experiences. This categorization is centered around two main categories of meditation styles: focused attention meditation (FAM) and open-monitoring meditation (OMM). FAM involves sustaining focus on a specific object (such as the breath or a specific bodily sensation), in this way enhancing concentration and attentional control (Lutz et al. 2008). An example of FAM is Samatha meditation. In OMM, on the other hand, the meditator engages in a non-specific monitoring of experiences, remaining attentive to any experience that might arise, without selecting, judging, or focusing on any particular object (Lutz et al. 2015; Tang et al. 2015). Vipassana meditation is generally assigned to this category. A number of studies have used this categorization to probe the neural correlates of meditation (Amihai and Kozhevnikov 2014; Hinterberger et al. 2014; Marzetti et al. 2014; Vyšata et al. 2014; Braboszcz et al. 2017; Kakumanu et al. 2018; Vivot et al. 2020; Yordanova et al. 2020; Calvetti et al. 2021; Young et al. 2021; Walter and Hinterberger 2022; D’Andrea et al. 2024).

Despite the growing body of research investigating neural criticality and complexity during the meditative state, the neural mechanisms underlying the different forms of meditation are still poorly understood, possibly due in part to the following factors: (i) many confounding variables, such as tradition, expertise, and control conditions, may be responsible for incoherent findings (Lutz et al. 2015; Tang et al. 2015); (ii) only a handful of studies have directly compared FAM and OMM (Vyšata et al. 2014; Kakumanu et al. 2018; Vivot et al. 2020; Young et al. 2021; Walter and Hinterberger 2022; D’Andrea et al. 2024; Ventura et al. 2024); and (iii) much of the literature is based on fMRI (Cooper et al. 2022), which lacks the temporal and spectral resolution needed to fully capture fine-grained modulations of complexity and criticality during meditation.

In the present study, we address these gaps by examining the neural dynamics of highly trained expert Buddhist monks performing Samatha (FAM) and Vipassana (OMM) meditation, as well as in a resting state (RS), by analyzing brain signals recorded with magnetoencephalography (MEG) (Arcara et al. 2023). Combining MEG’s high temporal resolution with measures of complexity and machine learning techniques, we test the hypothesis that, compared to RS, Samatha, and Vipassana, are associated with distinct changes in brain signal complexity and critical dynamics. To this end, we focus on a set of complexity and criticality-related measures, namely detrended fluctuation analysis (DFA) applied to both the broadband signal and the envelope of neural oscillations, the deviation from criticality coefficient (DCC), Lempel–Ziv complexity (LZC), spectral entropy (SpecEn), Higuchi’s fractal dimension (HFD), and the exponent of the aperiodic component of the power spectral density (PSD), also known as the 1/f slope. In tandem, we also explore changes in neural oscillations, whose role remains central to understanding brain dynamics. Previous studies have identified changes in power during meditation, particularly in the gamma range (Lutz et al. 2004; Cahn et al. 2010; Berkovich-Ohana et al. 2012). However, most of these studies did not distinguish between aperiodic and periodic components of the power spectrum, whose separation could potentially offer important insights into the underlying neural mechanisms (Donoghue et al. 2020a; Gerster et al. 2022; Lin et al. 2025). Given the evidence that the slope of the aperiodic component reflects the brain’s excitation–inhibition balance (Gao et al. 2017), we expect this aperiodic slope to change during meditation, potentially influencing interpretations of spectral power alterations. Specifically, as a secondary hypothesis, we explore whether the previously reported increases in gamma power may be partly explained by a broadband shift in the aperiodic component, rather than by enhanced oscillatory activity. To address this, we use the *specparam* (Donoghue et al. 2020b) method to separate aperiodic from periodic components of the PSD. This multifaceted approach provides an incisive and comprehensive understanding of how Samatha and Vipassana meditation modulate nonlinear brain dynamics.

## Materials and methods

To probe the large-scale brain dynamics associated with different types of meditation, we analyzed the neuromagnetic activity in a group of expert Buddhist monks during Samatha and Vipassana meditation. More specifically, we focused on changes in spectral power, complexity, and criticality-related measures.

### Participants

We recruited 12 professional meditators from the Theravada Buddhist tradition at the Santacittarama monastery in Italy. The participants were all male, with a mean age of 38.7 years (range 25–58 years, SD 10.9 years). The monks at Santacittarama follow a Theravada Thai Forest Tradition, which includes a 3-month winter retreat meditation and a balanced practice of Samatha and Vipassana. Outside the retreat period, monks communally practice Samatha–Vipassana meditation for 2 h daily, in addition to their own individual practice, maintaining always a balance between FAM and OMM. The participants recruited in the study had a combined meditative expertise of minimum 2375 h (mean meditation hours 15 343, range 2375–26 600, SD 8221).

### Procedure

The experimental paradigm consisted in a block design of 6-min Samatha meditation and 6-min Vipassana meditation blocks, each preceded and followed by 3 min of non-meditative resting state block (RS) (Marzetti et al. 2014). Each sequence was repeated three times, for a total of 12 sessions (6 RS, 3 Samatha, 3 Vipassana). For all conditions, subjects sat in the MEG scanner with eyes closed and did not employ any discursive strategy, recitation, breath manipulation, or visualization techniques. Data were recorded using a 165-channel MEG system installed inside a magnetically shielded room at the Institute of Advanced Biomedical Technologies, University of Chieti (Pizzella et al. 2001; Chella et al. 2012). This system includes 153 dc SQUID integrated magnetometers arranged on a helmet covering the whole head plus 12 reference channels. Electrocardiogram and electro-oculogram signals were also recorded for artifact rejection. All signals were band-pass filtered at 0.16–250 Hz and digitized at 1 kHz. Magnetic resonance images were acquired using a sagittal magnetization prepared rapid acquisition gradient echo T1-weighted sequence (MP-RAGE; Siemens Vision scanner 1.5 T; TR = 9.7 s, echo time TE = 4 ms, alpha = 12°, inversion time = 1200 ms, voxel size = 1 mm^3^). All procedures and protocols were approved by the Ethics Committee at the University of Chieti-Pescara, and were conducted in accordance with the local legislation and institutional requirements. The participants provided written informed consent, in accordance with the Declaration of Helsinki. The same dataset has been used in Marzetti et al. (2014) and D’Andrea et al. (2024) where more details on MEG data acquisition can be found.

### Data preprocessing and MEG source reconstruction

Following standard procedures, the raw MEG data were filtered with a zero-phase band-pass using a finite impulse response filter (FIR 1, order = 3, Hamming window) between 1 and 150 Hz and the filtered data were then downsampled to 600 Hz. Independent component analysis was used to remove eyeblinks, eye movements, heartbeat artifact, and external artifacts from the MEG signal. These analysis steps were performed using a standard preprocessing pipeline provided by NeuroPycon (https://github.com/neuropycon), an open-source Python package that provides template pipelines for advanced and fast multi-processing of neuroimaging data (Meunier et al. 2020). We excluded two subjects from further analysis due to excessive noise in the signal and missing MRI data. Each subject’s individual MRI data were segmented using the FreeSurfer software package (Fischl 2012) and was used to (i) generate a mixed source space that includes a discretization of both the cortical surface (8196 nodes with 5-mm voxel distance) and subcortical regions (Amygdala, Caudate, Cerebellum, Hippocampus, Thalamus); (ii) perform the co-registration between MRI and MEG devices; and (iii) compute the forward matrix using a Boundary Element Method algorithm with a single-layer model (Gramfort et al. 2011). Finally, for each subject, experimental condition (RS, Samatha, Vipassana), and session, we estimated the neural time series for each voxel in the mixed source space using the weighted Minimum Norm Estimate algorithm (Hämäläinen and Ilmoniemi 1994). The elemental dipoles of the cortical source-space were constrained to have an orientation normal to the surface, while the dipoles of the subcortical volumes were left with free orientation (Attal and Schwartz 2013). In total, after source-space morphing to a common MNI brain, we obtained 8196 time series at the cortical level and 3 time series per source in subcortical regions. We optimized computational efficiency by employing the source reconstruction pipeline of NeuroPycon, which runs the analyses on a multi-core processor.

### Features computation

To monitor the complexity and criticality-related dynamics of the monks’ brain activity while they were engaged in Samatha, Vipassana, and eyes-closed resting state, we extracted seven types of features from the MEG source time series: power spectral density (PSD) (both corrected and uncorrected for the 1/f spectral slope), the slope of the aperiodic component of the power spectrum, long-range temporal correlations (LRTC), deviation from criticality coefficient (DCC), Lempel–Ziv complexity (LZC), Higuchi fractal dimension (HFD), and spectral entropy (SpecEn). The following sections list the spectral, complexity, and criticality-related features we analyzed in this work. Unless otherwise specified, feature values were computed independently for each session and then averaged across sessions for each subject and condition.

### Power spectral density

We computed PSD at source level, in both cortical and subcortical regions, in the following frequency bands: delta (1–4 Hz), theta (5–7 Hz), alpha (8–12 Hz), beta (13–29 Hz), gamma1 (30–59 Hz), and gamma2 (60–90 Hz). We computed the PSD on the estimated neural time series using Welch’s method (Welch 1967) with a window length of 4 s and overlap of 2 s. Importantly, to isolate the spectral components that are strictly oscillatory in nature, we removed the aperiodic 1/f-like component from the power spectrum (see the next section) by subtraction. The 1/f-corrected spectrum was then divided into the same six frequency bands defined above.

#### Aperiodic slope

We used the specparam algorithm (formerly fooof) (Donoghue et al. 2020b) to estimate the slope of the aperiodic component of the estimated neural time series, which corresponds to the exponent of the 1/f-like distribution of the power spectrum. The specparam algorithm decomposes the log power spectra into a summation of narrowband gaussians (periodic oscillations) and a linear trend (aperiodic component) within a broad frequency range. We used a 1–90-Hz frequency range and limited the number of oscillatory peaks to 6. In the following analysis, we express the slope as the exponent *α* in 1/*f^α^*, such that an increase of the slope corresponds to a steeper decline in power. EEG modeling work has suggested that such steepening may be explained by an increase in global cortical inhibition (Gao et al. 2017, but see Brake et al. 2024).

#### Long-range temporal correlations

We investigated the temporal dynamics of neural oscillations and of the broadband signal by probing for the presence of long-range temporal correlations (LRTC) in the amplitude envelope of the oscillations in different frequency bands using Detrended Fluctuation Analysis (DFA) (Nikulin and Brismar 2005; Hardstone et al. 2012). DFA is a well-established method that measures the fluctuations of a signal as a function of a sliding time window at multiple scales. The original signal is integrated to create a cumulative sum series and split into windows of different sizes that are equidistant on a logarithmic scale. In each window, the linear trend is removed and the fluctuation function is defined as the average root-mean-square for each window size. The slope of the line fitting the fluctuation function in the log–log plot can be interpreted as an estimation of the Hurst exponent and gives an indication of the scaling behavior of the time series. It is often referred to as the self-similarity parameter and can take on a value between 0 and 1, where a value between 0 and 0.5 represents negative temporal correlation (i.e. anti-persistence of the signal in time), 0.5 represents a random (time series is uncorrelated) signal, and a value between 0.5 and 1 represents a positive temporal correlation. In a persistent signal, large signal values are likely to be followed by additional large signal values and small values are likely to be followed by more small values. Conversely, in an anti-persistent signal (negative correlation) large values are likely to be followed by small ones and small values are likely to be followed by large ones. Thus, if the LRTC exponent decreases from a value close to 1 down to a value closer to 0.5, then the temporal correlations are considered to be less persistent in time (i.e. they decay faster in time). We performed DFA on the broadband signal and on the amplitude envelope of the neural oscillations in delta, theta, alpha, beta, gamma1, and gamma2 bands. To this end, we filtered the estimated neural time series using a finite impulse response filter (FIR1, order = 3) and applied the Hilbert transform (Le Van Quyen et al. 2001; Foster et al. 2016) to extract the amplitude envelope. DFA was performed for each of the three conditions (RS, Samatha, Vipassana) in the time range of 5–50 s for delta and theta bands, 2–50 s for alpha, and 1–50 s for beta and gamma over consecutive windows (no overlap). To render the calculation of the DFA more robust, we concatenated the different sessions of each condition (Chen et al. 2002; Botcharova et al. 2015). As highlighted in Hardstone et al. (2012), DFA estimates for window sizes *>*10% of the signal length are noisier due to a low number of windows available for averaging (cf. Hardstone et al. 2012; Irrmischer et al. 2018a).

#### Deviation from criticality coefficient

Neural activity can at many scales be observed to spread as cascades through space and time, known as neuronal avalanches (O’Byrne and Jerbi 2022). At the critical point, neuronal avalanches exhibit scale invariance; i.e. the probability distribution of various avalanche properties, such as their size and duration, follow a power law, a mathematical relationship where one quantity varies as a power of another, resulting in a straight line when plotted in double-logarithmic axes (log–log). The slope of this line is the exponent of the power law and reflects the particular nature of the scale-invariant behavior. Unlike systems with characteristic scales, which are often well described by their mean and standard deviation, scale-invariant (or scale-free) phenomena are better characterized by this exponent, as it captures the particular relationship between successive scales (Hardstone et al. 2012).

While the detection of such power-law behavior provides an indicator of criticality, on its own it is not a very specific indicator, as other simple processes also yield such power-law scaling (Touboul and Destexhe 2017). However, criticality theory predicts that the exponents of these power laws should follow certain interrelations, known as scaling relations (Munoz et al. 1999; Friedman et al. 2012), that are not expected to be obeyed away from criticality (Touboul and Destexhe 2017). The degree of adherence to these relations is thus a fairly reliable indicator of the distance to criticality. One of these relations, known as the crackling noise relation (Sethna et al. 2001), connects the critical exponents for the distributions of avalanche size *S* (defined as the sum of events across all voxels, where an event is defined as a local maximum or minimum in the *z*-scored signal exceeding a data-driven threshold), avalanche duration *T*, and average *S* for every *T*:


$$ P\ (S)\propto{S}^{-\tau },P\ \left(T\ \right)\propto{T}^{-\alpha },<S>\left(T\ \right)\propto{T}^{1/\sigma \nu z} $$

which should be related as


$$ \frac{\alpha -1}{\tau -1} = \frac{1}{\sigma \nu z} $$

where *τ*, *α,* and *σνz* are the exponents of the power laws of these distributions and are known as critical exponents. We note that this relation holds generally for any class of criticality with an absorbing state (Munoz et al. 1999). From this relation, the deviation from criticality coefficient (DCC) (Ma et al. 2019) is defined as


$$ DCC=\frac{\alpha -1}{\tau -1}-\frac{1}{\sigma \nu z} $$

To detect neuronal avalanches, source-reconstructed time series were *z*-scored voxelwise and binarized according to a threshold determined using the following data-driven method (Shriki et al. 2013; Varley et al. 2020). The *z*-scored time series were first averaged across all subjects within the same condition and plotted in a probability distribution ranging from −10 to 10 SD. A Gaussian was fitted to this distribution and the binarization threshold was taken as the point where the Gaussian diverges from the data distribution, which represents the SD at which neural events are no longer expected to be the result of merely stochastic fluctuation. Following this method, the binarization threshold was set to *±*3 SD and negative and positive excursions beyond the threshold were identified as events and assigned a value of one at their maximum/minimum value. An avalanche was defined as a continuous sequence of events, on any voxel, separated in time by at most the time bin *t* = 4 ms (Shriki et al. 2013; Sorrentino et al. 2023). Avalanches were detected in this way for each subject and condition. The size, duration, and average size by duration distributions were plotted, and the critical exponents fitted from the distributions. We computed the DCC for each region of interest (ROI) defined by the Destrieux anatomical parcellation, which contains 74 anatomical regions in each hemisphere (Destrieux et al. 2010), using the *edgeofpy* Python package (https://github.com/jnobyrne/edgeofpy).

#### Lempel–Ziv complexity

Lempel–Ziv complexity (LZC) provides a measure of entropy by counting the number of distinct patterns of activity in the data (Le Van Quyen et al. 2001). It can be thought of as being proportional to the size of a computer file containing the data, after applying a compression algorithm (Schartner et al. 2015). LZC is a complexity measure designed for binary sequence and text and is computed on a binary version of the signal. The time series were binarized by using the median value: the values above the median are assigned ones, and below the median are assigned zeros. LZC is defined as the number of unique subsequences in the whole binary sequence. A less complex signal consists of repetitions of a few different sub-strings while more complex signals are made up of non-repeating segments. As the original version of LZC is strongly influenced by signal length, we used a normalized version which scales LZC by log*_b_*(*n*)/*n*, where *n* is signal length and *b* = 2 the number of unique characters in the signal (Zhang et al. 2009).

#### Higuchi fractal dimension

We used Higuchi’s algorithm (Higuchi 1988) to compute the fractal dimension of the estimated neural time series. From the original time series *X*, *k* new different time series *X_m_^k^*, *m* = 1, …, *k* are constructed and their average length *L*(*k*) is computed (*m* indicates the initial time sample and *k* denotes the time interval). This step is repeated for *k* = 1, …, *k*_max_. The average length *L*(*k*) is proportional to *k^−D^*, then plotting log(*L*(*k*)) against log(1*/k*) the slope *D* of the line fitting the points in the log–log plot represents the Higuchi fractal dimension (HFD). Numerical values of HFD have the lower and upper limits of 1 and 2, respectively. Higher values of the slope indicate a more complex curve, whereas values close to 1 indicate a simple curve with low complexity. HFD can be imaged as a measure of the “degree of filling out” of the plane by the curve and, hence, its complexity (Klonowski 2007). The value of *k*_max_, the maximum number of subseries composed from the original time series, was determined by examining the data and plotting the fractal dimension over a range of *k*. For *k* greater than *k*_max_, the fractal dimension reaches a saturation point (Kesić and Spasić 2016; Marino et al. 2019; Porcaro et al. 2022; Porcaro et al. 2024). Based on this procedure, we found the optimal *k*_max_ value to be 10. We also tested the robustness of the method with respect to the length of the time series and different values of *k*.

#### Spectral entropy

To quantify the complexity of the power spectrum, we computed the spectral entropy (SpecEn) (Pardey et al. 1996) by computing the Shannon entropy of the PSD of the neural time series. Shannon entropy (Shannon 1948) measures the uncertainty associated with a random variable and is calculated by taking the average value of the logarithm of the probability of each possible event. The power spectrum we used to compute SpecEn was estimated as described above, except that it was neither corrected for the 1/f-like component nor split into frequency bands. The spectral range used for entropy estimation was 1–90 Hz.

### Statistical analysis and machine learning

We examined the task-based modulations of all computed features (PSD, slope, LRTC, LZC, HFD, SpecEn) using (i) group-level differences assessed using cluster-based permutation tests (Maris and Oostenveld 2007) and (ii) a multi-feature supervised learning approach, where a Random Forest (RF) classifier was trained to discriminate between the different brain states in a *k*-fold cross-validation scheme (see below). We assessed the statistical differences in the DCC across the three conditions using the non-parametric Wilcoxon signed-rank test, applied to each ROI of the Destrieux parcellation.

#### Cluster-based permutation test

For each feature, statistical analysis was conducted to look for group-level differences (i) in each meditative state (Samatha, Vipassana) as compared to RS condition and (ii) between the two meditative states. Since for each subject we used an individual source space, before performing the statistical hypothesis test, we morphed and projected individual source space onto the average brain fsaverage provided by FreeSurfer.

Differences between the different conditions (RS, Samatha, Vipassana) for the several features were assessed using the cluster-based permutation tests corrected for multiple comparisons as implemented in MNE-python (Gramfort et al. 2014). The procedure uses a cluster analysis with permutation test for calculating corrected *P*-values. First, clusters are identified based on the spatial adjacency of voxels where the observed effect (difference between conditions assessed by a paired *t*-test) is statistically significant (*P*_val_ *< .*05). Then, each cluster is associated with a cluster-level statistic (the sum of voxel *t*-values within the cluster) that is compared to the null distribution of 1024 permutations using shuffled labels. Differences between conditions are considered significant when the observed cluster-level statistic exceeds the maximum cluster-level statistics from the null distribution using a threshold of *P*-value = .05 corrected for multiple comparisons (Maris and Oostenveld 2007).

#### Pearson correlation analyses

We evaluated the relationship between hours of meditation practice and the features where we found a state effect induced by the meditative condition. Specifically, we calculated the relative difference (M − RS)/RS of the features, where M is either Samatha or Vipassana. This was computed within voxels of the clusters determined to be significant in the previous analysis. Individual values in these clusters, for each subject, were averaged across each cluster and then used to calculate their Pearson correlation with the expertise of each monk. To this end, we used the log of the total hours of meditation, as in previous reports (Braboszcz et al. 2017). For the DCC feature, Pearson correlations were computed by averaging, for each subject, the DCC values within the ROIs that showed significant difference between conditions. To control for multiple comparisons, we applied false discovery rate (FDR) correction to the Pearson correlations using the Benjamini–Hochberg procedure (Haynes 2013). Additionally, to explore the relations between the different measures, we computed, for each meditation condition, the Pearson correlation between feature pairs, considering for each pair the average values across ROIs. For the DCC feature, we also inspected, for each ROI of the Destrieux atlas, the correlation between the DCC and all other features.

#### Multi-feature classification

We additionally implemented a multi-feature classifier to characterize the contribution of the different features in discriminating between meditative states and resting-state condition. To identify which brain regions contribute more to the separation, we reduce each feature (except for the DCC, which already provides a single value for ROI) to a low dimensional space by computing the mean values over the ROIs provided by the 148-label of the Destrieux parcellation and the 10 subcortical regions. Then, we took together all features from all ROIs to construct a single model and we were able to access the contributions of individual features and ROIs by examining feature importance. To achieve this, we used a random forest (Breiman 2001) classifier, trained on all extracted features (18 measures × 158 ROIs = 2844 features). The model estimates feature importance by the relative rank of each feature across the decision trees that make up the forest. To measure variance in feature importance, the training process was repeated 120 times for each comparison (Samatha versus RS, Vipassana versus RS, Samatha versus Vipassana), applying grid search with grouped 7-fold cross-validation inside a nested cross-validation, leaving out samples from three subjects in each iteration. The overall model score was determined using samples from left-out testing subjects and the area under the curve (AUC) was used to evaluate the performance of the classifier (Thölke et al. 2023).

## Results

The following section describes the results obtained by contrasting the three different conditions (RS, Samatha, and Vipassana). We start by reporting spectral data, which provide insight on changes in periodic brain activity; then, we describe the results of non-parametric statistical tests for each of the extracted features (narrowband power, 1/f slope, LRTC, LZC, HFD, SpecEn, and DCC) and the output of the multi-feature classifier. Except for DCC (Fig. 5), the direct comparison between Samatha and Vipassana did not yield significant differences. The summary of these findings for the non-significant features (narrowband power, 1/f slope, LRTC, LZC, HFD, SpecEn) is provided in the supplementary material (Fig. S1).

### Meditation induces changes in MEG oscillations

**Figure 1:**
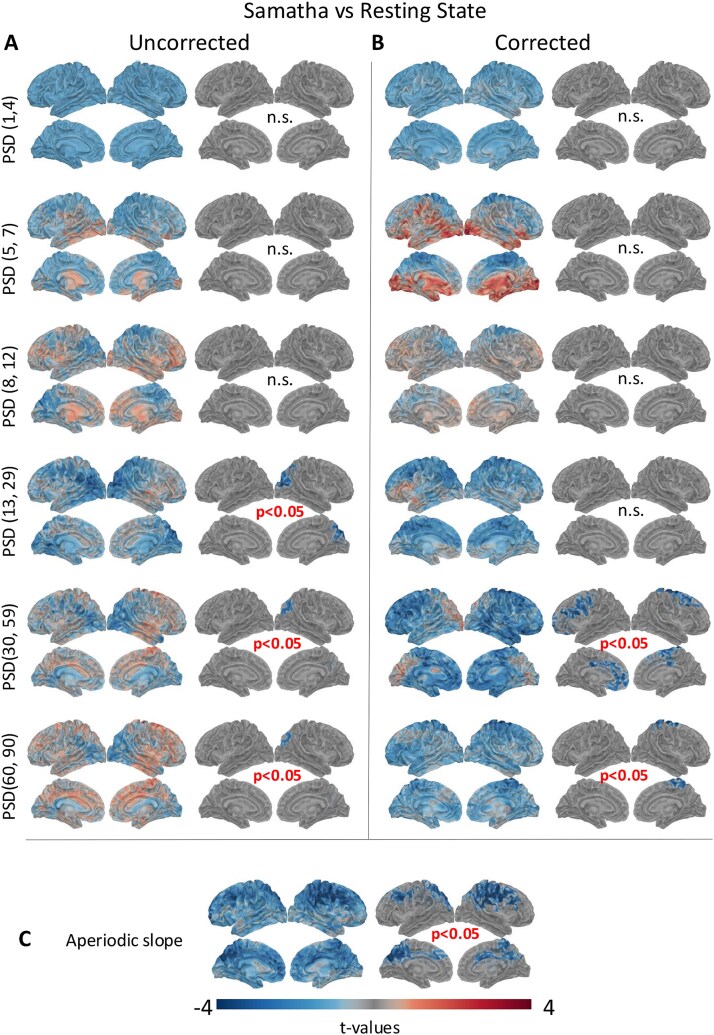
Results of the source-level spectral power analysis in the different frequency bands for Samatha as compared to RS condition. (A) The left column shows the results of two-tailed paired *t*-tests, and the right column shows the results of cluster-based permutation tests (*P < .*05). (B) *t*-Maps and results of cluster-based permutation tests for spectra corrected for the 1/f slope. (C) *t*-Maps and results of cluster-based permutation tests for the aperiodic component (1/f slope) of the power spectra.

**Figure 2:**
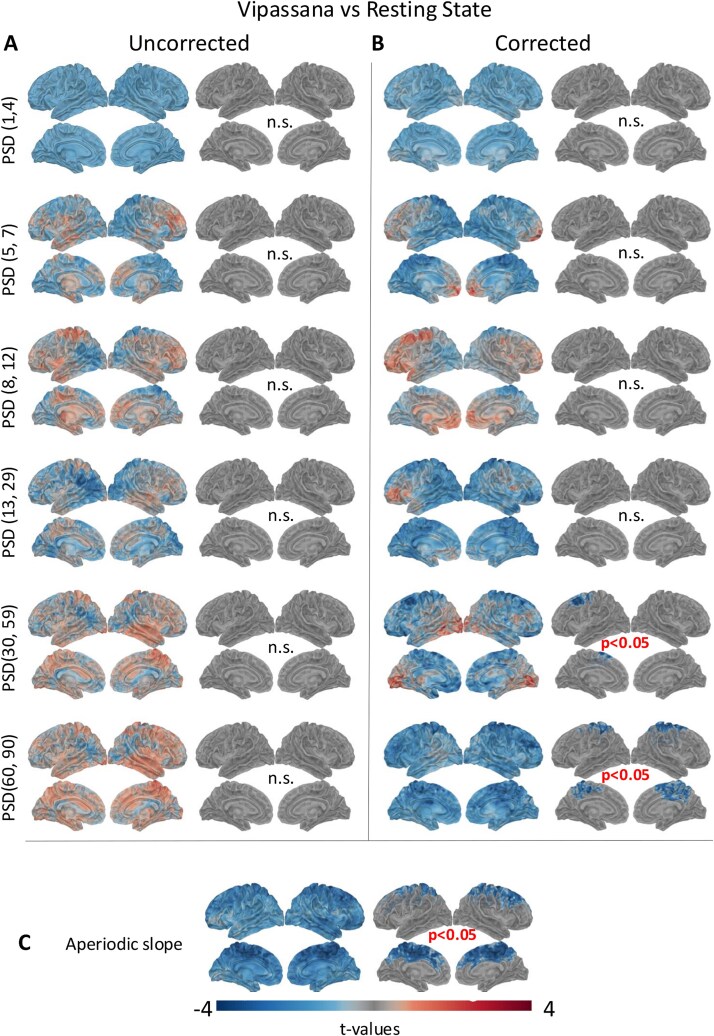
Results of the source-level spectral power analysis in the different frequency bands for Vipassana as compared to RS condition. (A) The left column shows the results of two-tailed paired *t*-test, and the right column shows the results of cluster-based permutation tests (*P* < .05). (B) *t*-Maps and results of cluster-based permutation tests for spectra corrected for the 1/f slope. (C) *t*-Maps and results of cluster-based permutation test for the aperiodic component (1/f slope) of the power spectrum.

Figures 1A and 2A show the results of the source-level spectral power analysis by frequency band, for Samatha versus RS and Vipassana versus RS, respectively. The left column shows the spatial distribution of *t*-values obtained by two-tailed paired *t*-tests, while the right column displays only the *t*-values that were significant (*P < .*05) based on cluster-based permutation tests. Samatha meditation led to a statistically significant reduction of power (*P < .*05) in beta, gamma1, and gamma2 bands, mainly in occipital and parietal regions.

Interestingly, the spectral results are quite different when we correct for the aperiodic slope (Figs. 1B and 2B). Because the power spectrum is the sum of peaks of oscillatory (periodic) activity and a background of non-oscillatory (aperiodic) frequency content (Donoghue et al. 2020b), not performing the correction may lead to confounding results that conflate oscillatory and non-oscillatory signals (Donoghue et al. 2020a; Lin et al. 2025; Rodriguez-Larios et al. 2021). With the aperiodic component removed in our data, we note that the direction of the effect is consistent for all frequency bands, with the exception of the beta band for Samatha and the gamma bands for Vipassana. Discarding the aperiodic component reveals that both meditation practices induced a statistically significant reduction in gamma power (*P < .*05), especially in the precentral and superior frontal regions (Figs. 1B and 2B right column).

Figures 1C and 2C show the *t*-maps and the results of cluster-based permutation tests for the aperiodic slope for Samatha versus RS and Vipassana versus RS, respectively. We found a statistically significant reduction of the slope (*P < .*05) in both meditative conditions, mainly in the frontal, parietal, occipital, and temporal regions. This finding supports our choice to perform the correction. Such changes in the scaling behavior of the power spectra suggest meditation-induced changes in self-similarity of the electrophysiological signal, which may be related to an alteration in neural criticality. In addition, previous work supports the notion that a reduction in the 1/f exponent (i.e. flatter slope) may reflect an increase in the E-I ratio.

### Meditation alters complexity and criticality-related measures

**Figure 3:**
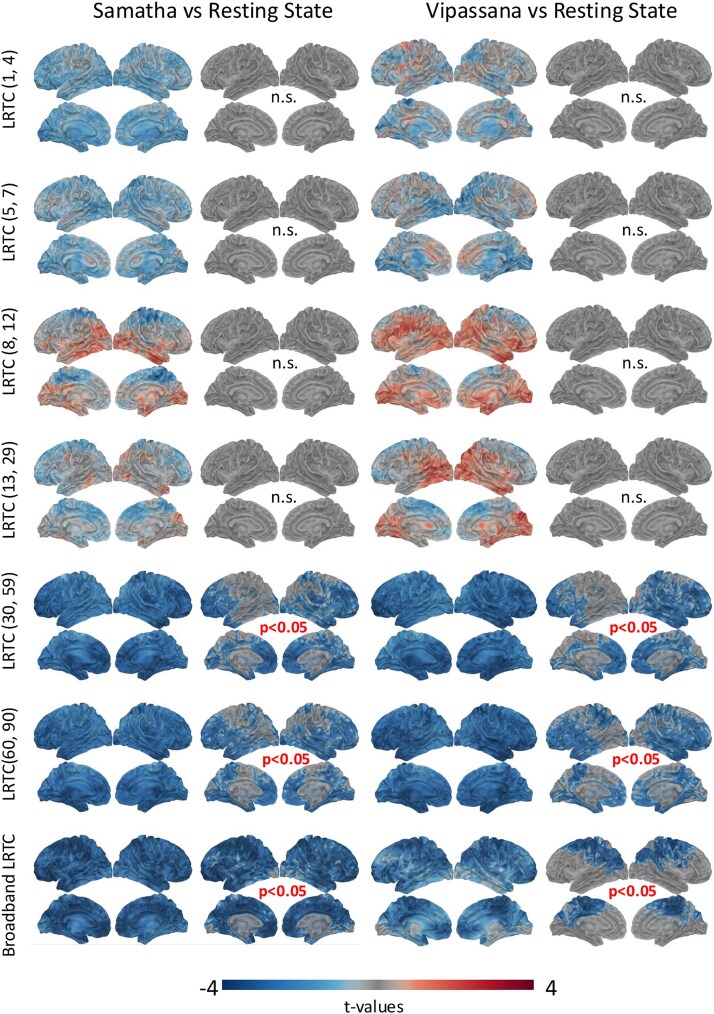
*t*-Maps and results of cluster-based permutation tests for the LRTC features when the two meditation conditions (Samatha on left, Vipassana on right) are compared to the RS condition.

We investigated the effects of meditation on the temporal persistence of neural oscillations by computing the LRTC in different frequency bands (delta, theta, alpha, beta, gamma1, and gamma2) and on the broadband signal. Across conditions, we consistently found values for the LRTC exponent *>*0*.*5, indicating a persistent, positively correlated signal. The *t*-maps obtained by the two-tailed paired *t*-tests and the results of the cluster-based permutation tests (*P < .*05) are shown in Fig. 3 for Samatha (left) and Vipassana (right). The *t*-value maps reflect the magnitude of the effect and the direction of the group differences for the LRTC values, with negative (blue) *t*-values indicating the brain areas where meditation induced a reduction of the LRTC. Marked decreases in LRTC were found across the cortex for gamma1 and gamma2 bands, as well as for the broadband signal, in both meditation conditions. The reduction in LRTC values reflect weaker intrinsic fluctuations in neural activity indicating diminished signal memory and weaker persistence of past neural states. This result is in line with the decreasing slope of the aperiodic activity (Herzog et al. 2024; Thölke et al. 2025): the combination of weaker LRTC (less signal memory) and a more excitable network (flatter PSD slope) may support a state of cognitive adaptability (Herzog et al. 2024), consistent with the demands of meditative attention.

In addition, we found a decrease of DCC (an indicator of the distance from the critical point) from RS to Vipassana and from Samatha to Vipassana. This decrease was statistically significant (*P <* .05, uncorrected) in specific ROIs of frontal, parietal, occipital, temporal, and insular regions, which largely correspond to areas within the Ventral Attention, Dorsal Attention, Motor, and Default Mode Networks (Fig. 5). We point out that these effects did not pass the correction for multiple comparisons and should therefore be interpreted as trend results that require further investigation. This being said, the unanimously consistent direction of the observed trends of decreasing DCC lends them heavier credence.

**Figure 4:**
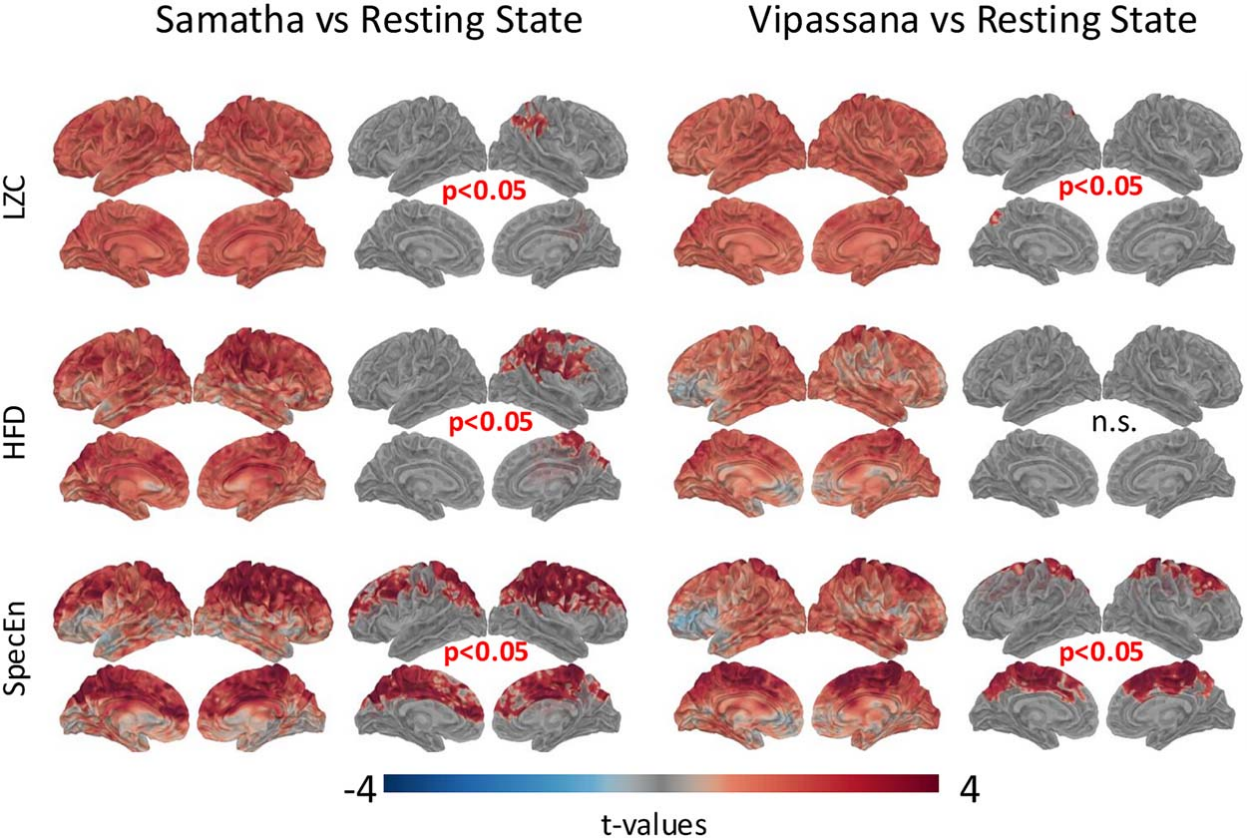
*t*-Maps and results of cluster-based permutation tests for the complexity features (LZC, HFD, and SpecEn) when the two meditation conditions (Samatha on left, Vipassana on right) are compared to the RS condition.

The LZC and HFD *t*-maps show an overall increase in both metrics (Fig. 4) for the two meditation conditions as compared to RS. In particular, we note a significant increase (*P < .*05) of LZC in Samatha mainly in parietal and temporal regions of the right hemisphere, and in Vipassana in precuneus and parietal regions of the left hemisphere. For HFD, the results of cluster-based permutation tests reveal a significant increase (*P < .*05) only for Samatha in the right hemisphere, mainly in the precentral, postcentral, superior temporal, and frontal regions.

**Figure 5:**
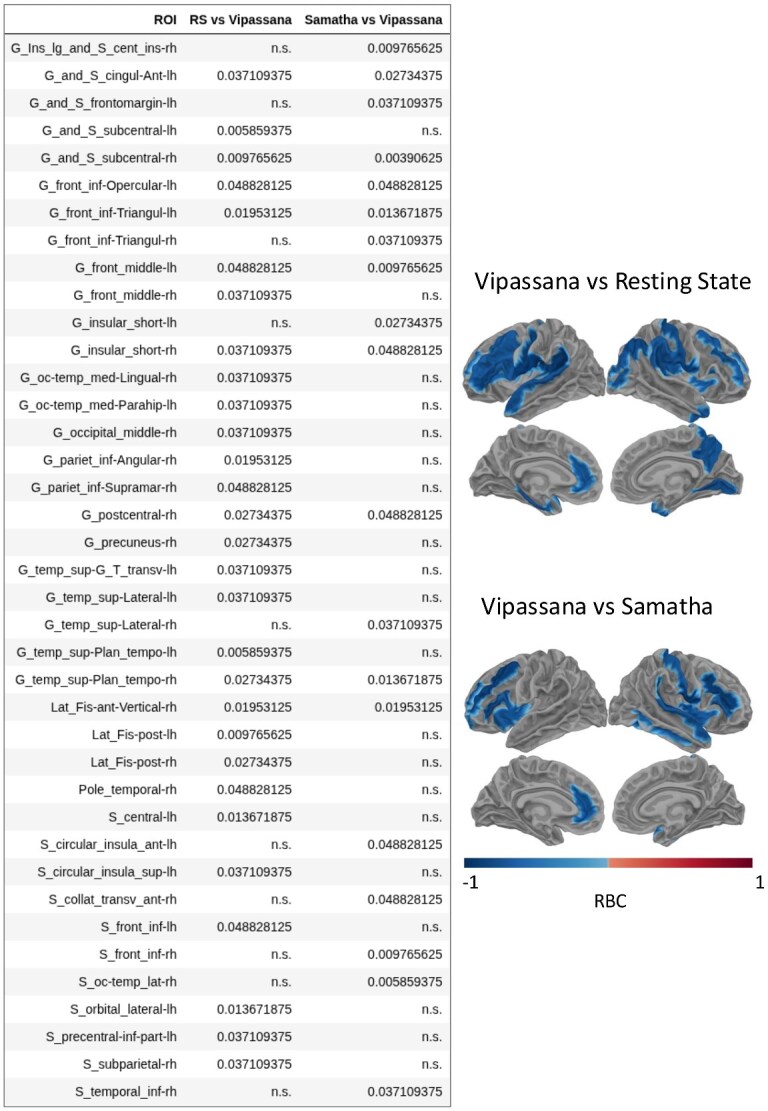
Left: Table of uncorrected *P*-values (*P* < .05) from the non-parametric Wilcoxon signed-rank test for each ROI in the Destrieux atlas, assessing pairwise comparisons of the deviation from criticality coefficient (DCC) across the three conditions. Right: Brain plots showing rank biserial correlation (RBC) values for the same pairwise comparisons. RBC quantifies the effect size as the difference between the proportion of favorable evidence minus the proportion of unfavorable evidence with values ranging from −1 to 1: positive values indicate higher DCC in the first condition, and negative values reflect a decrease. Only ROIs with statistically significant differences (*P* < .05 uncorrected) are displayed.

**Figure 6:**
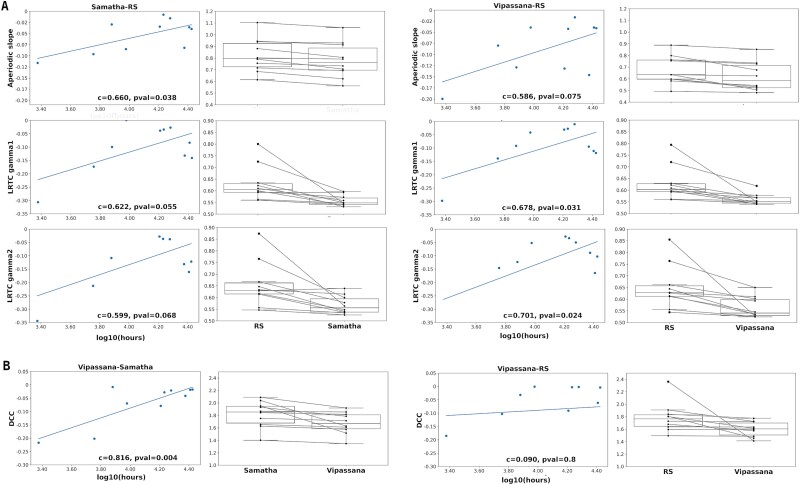
Correlations with hours of meditation and box plots, by feature. (A) The first and third columns show the Pearson correlation between hours of meditation and the features in which we found a state effect of Samatha and Vipassana, respectively. The second and fourth columns illustrate the box plot of individual features values (averaged within the significant clusters) for Samatha and Vipassana, respectively. (B) The first and third columns show the Pearson correlation between hours of meditation and the DCC feature. The second and fourth columns illustrate the box plot of DCC values, averaged within the ROIs that showed significant differences between conditions.

**Figure 7:**
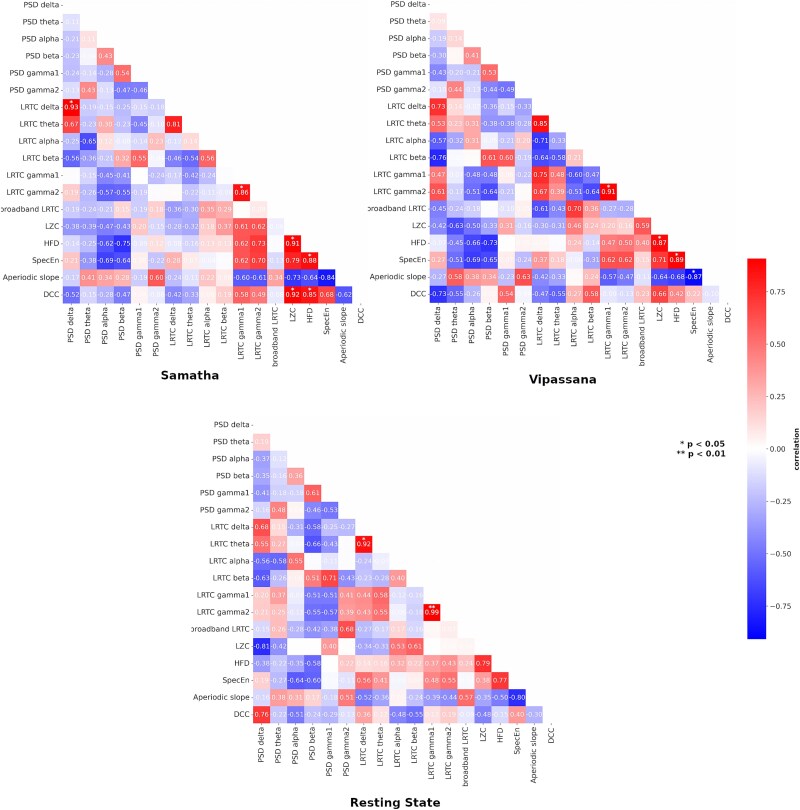
For each condition (resting state, Samatha, and Vipassana), correlation matrix between power, DCC, all complexity, and criticality-related measures. *P*-values were corrected using false discovery rate (FDR) correction.

**Figure 8:**
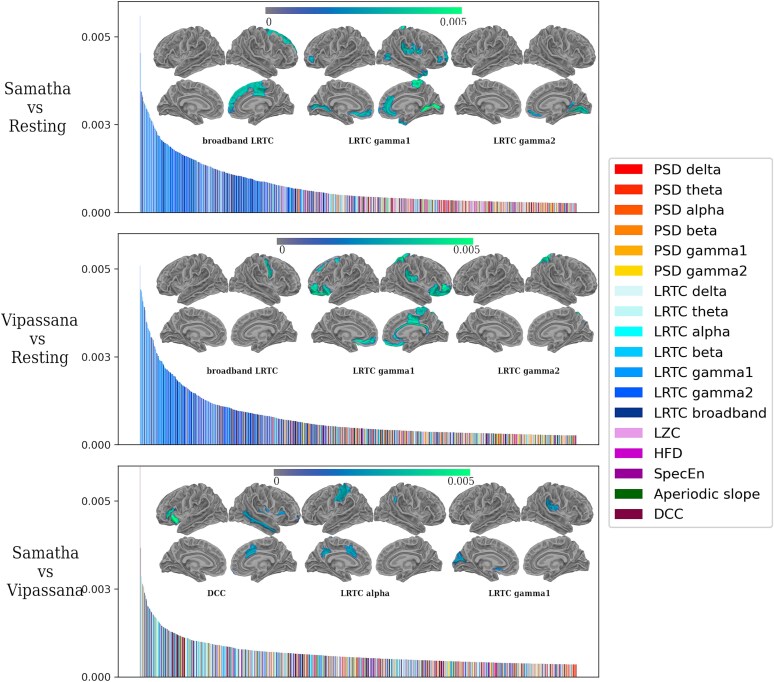
Visualization of ranked feature importance for the random forest classifiers trained on a feature space which combined all measures across ROIs (18 measures × 158 ROIs = 2844 total), color coded according to the corresponding measure. Warm colors (red to yellow) show spectral power in the different frequency bands, while the other colors (blue, violet, and green) correspond to complexity and criticality-related measures. The cortical maps depict the spatial distribution of 20 most important features. Here, we show the average feature importance over different runs: higher values indicate higher importance of a feature at a specific ROI.

Finally, the SpecEn feature (Fig. 4) quantifying the complexity of the power spectra significantly increased (*P < .*05) in both meditation conditions, mainly in the cingulate, central, and frontal regions.

### Correlation analysis

Figure 6A shows both the correlations between the computed features and the hours of meditation, as well as the box plots for the values of the individual features (averaged within the significant clusters) for both main comparisons: Samatha versus RS and Vipassana versus RS. Here, we report only those correlations that showed a trend-level effect, for which uncorrected *P*-values *<*.05. Specifically, for Samatha the number of meditation hours was positively correlated with the aperiodic slope (*P* = .038) while Vipassana meditation showed a positive correlation with the LRTC for gamma1 (*P* = .031) and gamma2 (*P* = .024). These trends indicate that more experienced meditators may exhibit smaller differences between rest and meditation, suggesting that with increasing expertise, brain dynamics during meditation become more similar to those at rest. Figure 6B shows the result of correlation analysis for the DCC feature, revealing a positive correlation between the Vipassana–Samatha comparison and meditation hours (*P* = .004), indicating that the difference between the two meditation styles diminishes in highly experienced practitioners.

To rule out age as a confounding factor, we performed partial correlation analyses using age as a covariate. All previously trend-level associations remained significant (for Samatha–RS aperiodic slope: *c* = 0.57, *P* = .05; LRTC gamma1: *c* = 0.64, *P* = .03; LRTC gamma2: *c* = 0.59, *P* = .046; for Vipassana–RS LRTC gamma1: *c* = 0.58, *P* = .05; LRTC gamma2: *c* = 0.6, *P* = .043; for Vipassana–Samatha DCC: *c* = 0.75, *P* = .009), suggesting that the relationships between meditation experience and some features are not solely driven by age. These results should nonetheless be interpreted with caution due to the small sample size and the fact that none of the effects remained significant after correction for multiple comparisons.

The boxplots (Fig. 6, S2) show, for both meditation conditions, a local state effect characterized by a decrease in spectral features (PSD gamma1, PSD gamma2) and criticality-related measures (aperiodic slope, LRCT gamma1, LRCT gamma2, broadband LRTC), as well as an increase in complexity measures (LZC, HFD, and SpecEn). DCC values decrease from RS to Vipassana and from Samatha to Vipassana, possibly reflecting a shift in brain dynamics toward a more critical regime during meditation practice.

Finally, Fig. 7 shows, for each condition, the correlation matrix between feature pairs. Correlations marked with ^*^ correspond to a *P*-value *<*.05, as corrected for multiple comparisons using FDR. In both meditation types, we noted a consistent negative correlation between the aperiodic slope and all three complexity measures (LZC, HFD, SpecEn), indicating that as the aperiodic slope becomes steeper the overall brain signal complexity tends to decrease. Surprisingly, in Samatha, DCC showed strong positive correlations with the complexity measures (LZC: *r* = 0.92; HFD: *r* = 0.85; SpecEn: *r* = 0.68), a positive correlation with high-frequency LRTC (LRTC gamma1: *r* = 0.58; LRTC gamma2: *r* = 0.49), and a negative correlation with the aperiodic slope (*r* = −0.62), counterintuitively indicating that a greater deviation from avalanche criticality is associated with increased brain signal complexity, enhanced LRTC, and flatter slope. This pattern is further supported by ROI-level correlation analysis (Fig. S3), which reveals a positive correlation between DCC and complexity measures across different ROIs, suggesting a widespread, rather than localized, engagement of brain regions in this DCC–complexity/slope correlation during Samatha meditation. In contrast, Vipassana showed weaker correlations between DCC and complexity measures (LZC: *r* = 0.66; HFD: *r* = 0.42; SpecEn: *r* = 0.22), and a negligible correlation between DCC and both the high-frequency LRTC (LRTC gamma1: *r* = −0.09; LRTC gamma2: *r* = −0.13) and aperiodic slope (*r* = −0.1). The widespread strong correlation between DCC and complexity/slope observed for Samatha is not present for Vipassana (Fig. S3).

### Multi-feature machine learning

We built a Random Forest classifier that we trained on all extracted features (18 measures × 158 ROIs = 2844 total) giving us an insight into individual feature importance scores by ranking and comparing the contributions of the different features in discriminating between meditative states and RS condition. For the PSD features, we considered only those corrected for the aperiodic component. We found that the multi-feature RF classifier achieved a higher decoding accuracy for Samatha versus RS (score = 76.53 *±* 0.14, AUC = 90.23 *±* 0.1) and Vipassana versus RS (score = 73.06 *±* 0.14, AUC = 87.18 *±* 0.12) than for Samatha versus Vipassana (score = 48.06 *±* 0.14, AUC = 49.03 *±* 0.15). To assess the significance of the obtained AUCs, we performed a permutation test for each comparison by shuffling the feature labels 100 times and computing the 95th percentile of the null distribution. We found that observed AUC values for Samatha versus RS and Vipassana versus RS were statistically significant.

Figure 8 displays the ranked feature importance, colored according to the feature category. The features of highest importance, appearing toward the left-hand side of the plots, were the ones most relevant in discriminating between the different brain states. For Samatha and Vipassana versus RS, the most important features were the LRTC in gamma1 and gamma2 bands and in the broadband signal (in blue). For Samatha versus Vipassana, DCC was the feature that most contributed to the discrimination together LRTC in alpha and gamma1. The cortical maps (inset) represent the spatial distribution of the 20 most important measure–ROI pairs (all of which happen to be in broadband LRTC, LRTC alpha, LRTC gamma1, LRTC gamma2, and DCC). Here, we show the average feature importance over different runs: higher values indicate higher importance of a measure at a specific ROI. The ROIs that contributed the most in classifying the meditation conditions versus RS were located mainly in the frontal, anterior cingulate, lingual, and insular regions for Samatha, and in the superior frontal, precentral, and parietal regions for Vipassana. Table 1 provides a list of these 20 important measure–ROI pairs as determined by the RF classifier. Overall, these results highlight LRTC, a criticality-related measure, as highly discriminative between meditation and RS, as well as between different meditation styles.

**Table 1:** List of the 20 most important measure–ROI pairs as determined by the Random Forest classifier

**Samatha versus resting state**		
**Feature**	**ROI**	**Importance**
LRTC gamma1	S_calcarine-rh	0.005593
LRTC gamma1	G_and_S_paracentral-rh	0.004539
LRTC gamma2	G_oc-temp_med-Lingual-rh	0.003441
Broadband LRTC	G_and_S_cingul-Mid-Post-rh	0.003431
LRTC gamma1	S_suborbital-lh	0.003390
Broadband LRTC	G_front-sup-rh	0.003332
Broadband LRTC	S_front-sup-rh	0.003287
LRTC gamma1	G_and_S_cingul-Ant-rh	0.003174
LRTC gamma1	G_and_S_subcentral-rh	0.003159
LRTC gamma1	Lat_Fis-ant-Horizont-rh	0.003131
LRTC gamma1	S_occipital-ant-rh	0.003102
LRTC gamma1	G_subcallosal-lh	0.003062
LRTC gamma1	G_and_S_transv_frontopol-rh	0.003040
LRTC gamma1	Lat_Fis-post-rh	0.003033
LRTC gamma1	S_orbital_lateral-rh	0.002957
LRTC gamma1	S_orbital_lateral-lh	0.002953
LRTC gamma2	G_cingul-Post-ventral-rh	0.002937
LRTC gamma2	S_suborbital-lh	0.002907
LRTC gamma1	Pole_temporal-rh	0.002869
LRTC gamma1	S_calcarine-lh	0.002844
**Vipassana versus resting state**		
**Feature**	**ROI**	**Importance**
LRTC gamma1	S_orbital_lateral-rh	0.005088
LRTC gamma1	G_orbital-lh	0.004425
LRTC gamma1	G_subcallosal-lh	0.004406
LRTC gamma1	G_parietal-sup-rh	0.004351
LRTC gamma2	G_parietal-sup-rh	0.004230
LRTC gamma1	G_and_S_frontomargin-rh	0.004190
LRTC gamma1	S_suborbital-lh	0.004089
LRTC gamma1	S_orbital_lateral-lh	0.004064
LRTC gamma1	S_pericallosal-rh	0.003939
LRTC gamma1	S_central-rh	0.003917
Broadband LRTC	S_central-rh	0.003911
LRTC gamma1	S_orbital-H_Shaped-lh	0.003842
LRTC gamma1	G_and_S_cingul-Mid-Post-rh	0.003822
LRTC gamma1	G_rectus-rh	0.003599
LRTC gamma1	Lat_Fis-post-rh	0.003496
LRTC gamma1	S_circular_insula-ant-lh	0.003480
LRTC gamma1	S_front_sup-lh	0.003446
LRTC gamma1	S_circular_insula-ant-lh	0.003441
LRTC gamma1	G_S_paracentral-rh	0.003317
LRTC gamma1	S_orbital-H_Shaped-rh	0.003278

## Discussion

While meditation continues to garner widespread interest and becomes more integrated into therapeutic practices, the electrophysiological changes it induces in the brain remain poorly understood. Yet, a better understanding of the neural dynamics underlying distinct meditation techniques is needed in order to tailor interventions for specific psychological conditions, optimize therapeutic outcomes, and refine methodologies for training and practice. Here, we analyzed rare neuromagnetic data recorded in expert meditators while they performed two types of meditation known as Samatha and Vipassana. Our analysis offers a novel contribution by combining source-space MEG analyses with the use of cutting-edge methods from dynamical systems theory, complexity science, spectral analyses, and machine learning. First of all, we examined the effect of separating periodic and aperiodic components of the power spectrum on meditation-induced spectral power changes. This allowed us to probe whether previously observed increases in gamma power during meditation could in fact be attributed to a broadband upward shift in the aperiodic component in the higher frequencies, rather than genuine increases in gamma oscillation amplitude. Second, we focused on characterizing changes in nonlinear brain dynamics induced by the different meditation techniques through the lens of signal complexity and criticality-related metrics. In the subsequent sections, we delineate our principal findings, contextualizing them within the framework of existing literature. Additionally, we acknowledge the constraints of our study and outline prospective directions for further research.

### Meditation induces changes in spectral power and 1/f slope

Our findings on power spectral density reveal a reduction in the beta-band power for Samatha, in line with other studies using experienced meditators (Saggar et al. 2012; Amihai and Kozhevnikov 2014; Hinterberger et al. 2014; DeLosAngeles et al. 2016), which may reflect increased cortical activation of sensory-related attentional networks and an increased capacity of the expert meditators to focus their attention on a particular sensation, such as their breath.

Interestingly, when we used 1/f-corrected power spectra (i.e. excluding the aperiodic component), the results revealed that, compared to rest, both meditation conditions exhibited a decrease in oscillatory gamma power—an intriguing finding, given previous reports of increases in gamma-range activity during meditation (see discussion below).

High-frequency gamma oscillations have been shown to be involved in attentional mechanisms subserving visual processing and working memory (Jensen et al. 2007; Jokisch and Jensen 2007). Therefore, the decrease in oscillatory gamma power we found here could reflect a reduction in the mental activity that arises from processing external stimuli and engaging diverse cognitive processes. This aligns with the meditative goal of achieving a state of mental calm and inward focus. The brain areas in which we observed a statistically significant reduction are mainly the frontal, parietal, and cingulate regions, which are key regions in several brain networks, including the Frontoparietal, Dorsal Attention, Motor, and Default Mode networks (Yeo et al. 2011). Interestingly, the observed reduction in gamma power across these brain regions is putatively consistent with previous reports of decreases in fMRI activation in the Default Mode network (Garrison et al. 2015), and with the Topographic Reorganization Model of Meditation proposed by Cooper and colleagues (Cooper et al. 2022). The proposed framework, based primarily on fMRI findings, explains how advanced meditation reorganizes brain topography promoting a shift away from cognitive engagement toward a state of integrated awareness, associated with the state.

The observed decrease in oscillatory gamma power presents an interesting contrast with previous studies in experienced meditators (Lutz et al. 2004; Cahn et al. 2010; Berkovich-Ohana et al. 2012; Amihai and Kozhevnikov 2014; Walter and Hinterberger 2022; Merlet et al. 2024), which reported an increase in gamma both globally and locally in the frontocentral and posterior regions. A likely explanation for this discrepancy lies in the fact that these studies did not account for possible modulations of non-oscillatory components of the signal. Indeed, the use of power spectral density as a measure of EEG or MEG oscillations requires caution (Donoghue et al. 2020a; Merlet et al. 2024; Lin et al. 2025), as the power spectrum reflects not only oscillatory (periodic) brain activity but also the level of non-oscillatory (aperiodic) brain activity, which is captured by the 1/f slope of the spectrum (Donoghue et al. 2020b). Recent work (Donoghue et al. 2020b) on simulated and real data has highlighted how traditional band-limited power and band ratio measures can conflate these distinct neural components, complicating interpretation of observed changes.

Of course, in the absence of significant changes in the aperiodic component, estimating oscillatory power with or without correcting for the 1/f slope would not have much impact. However, as shown by our analyses, both meditation techniques showed a statistically significant reduction (i.e. flattening) of the slope compared to resting state. While these results do not falsify previous observations, they do suggest, in line with emerging research on the physiological meaning of the aperiodic slope (Donoghue 2024), that the reported increase of gamma power in other studies may, at least in part, be attributable to an upward broadband shift across higher frequencies due to aperiodic changes, rather than solely reflecting an increase in actual gamma oscillation amplitude. The disambiguation between the periodic and aperiodic changes induced by meditation, particularly in the high-frequency range, is crucial when it comes to the mechanistic interpretation. Our results underscore the relevance of considering the aperiodic component (i.e. the 1/f slope) of the power spectrum as a distinct and important brain feature modulated by meditation. As discussed in recent reviews specific to contemplative neuroscience (Lin et al. 2025), and in broader neurophysiological contexts (Donoghue et al. 2020b; Gerster et al. 2022), analyzing the aperiodic component offers complementary and potentially more precise insights into how meditation influences brain activity. A recent review by Lieberman et al. (2025) on the neurophysiological mechanisms of focused attention meditation highlights that a factor often overlooked in the interpretation of spectral power changes is the contribution of aperiodic activity. They argue that explicitly modeling the aperiodic component can help distinguish whether increased power in a specific frequency band during focused attention reflects enhanced neural synchrony or rather a broadband shift associated with underlying cognitive state changes. While we do not claim that calculating PSD without aperiodic correction is inherently incorrect, our findings suggest that future investigations of spectral power modulations under meditation could significantly benefit from examining the effect of explicitly characterizing and accounting for the aperiodic component (Donoghue et al. 2020b), thus providing a more nuanced and comprehensive understanding of neural changes. We also acknowledge that additional work, including more rigorous analyses and modeling, possibly using simulated data, is needed to fully clarify the specific contributions and interpretive value of aperiodic-corrected spectral features.

To the best of our knowledge, there is only one meditation study to date that has investigated changes in the slope of the power spectrum (Rodriguez-Larios et al. 2021). The authors compared EEG spectral modulations associated with meditation and mind wandering between experienced meditators and a control group without meditation experience. Their findings indicate that experienced meditators showed a non-significant increase in the steepness of the 1/f slope (i.e. more negative values) during uninterrupted breath-focused meditation compared to rest. This discrepancy between their results and ours could be attributed to methodological parameters including their use of a 19-channel EEG cap and their focus on a more limited frequency range (2–30 Hz) for EEG power spectrum, as well as potential discrepancies in the parameters used for the estimation of the spectral slope.

A tentative hypothesis for the observed reduction in the 1/f slope across the frontal, parietal, occipital, and temporal regions is that it may reflect alterations in the excitation-inhibition (E-I) ratio. Indeed, modeling work suggests that the aperiodic slope reflects changes in E:I balance (Gao et al. 2017) with a reduction in the 1/f slope (i.e. its scaling exponent) reflecting an increase in the E:I ratio. Interestingly, we observed a stronger drop in the 1/f slope for Vipassana as compared to Samatha. Moreover, these changes in the scaling behavior of the power spectra are linked to changes in self-similarity, which in turn can be related to shifts in neural criticality. Notably, E:I balance has been proposed as a control parameter that can drive neural dynamics closer to or further from the critical point, a state poised between order and chaos that allows for maximal computational efficiency and flexibility (O’Byrne and Jerbi 2022). A shift toward increased excitation has therefore been interpreted as moving closer to the critical regime (Thölke et al. 2025).

### Meditation effects on complexity and criticality-related measures

The DFA revealed prominent statistically significant decreases in the LRTC during Samatha and Vipassana meditations within gamma1 (30–59 Hz) and gamma2 (60–90 Hz) frequency bands and the broadband signal. A reduction in LRTC generally suggests a decrease in the persistence or memory of the system (reduced information propagation over time), suggesting changes in the underlying dynamical processes or a shift toward more random or less correlated neural activity. Irrmischer et al. (2018a) showed that meditation practitioners had weaker LRTC during a focused attention meditation compared to rest, based on DFA of EEG data. Interestingly, this reduction was not seen in participants with no meditation experience. The authors interpreted the results as a shift toward a subcritical regime and argued that the reduced autocorrelation within the signal may be associated with fewer distractions from the task and interpreted as a down-regulation of certain mental processing activities. Similarly, Walter and Hinterberger (2022) found that experienced meditators showed a significant reduction in the LRTC of broadband signal under three different meditation conditions (FAM, presence monitoring, and thoughtless emptiness) compared to the resting state. Our results are not only consistent with the previous findings but also suggest that the changes in LRTC, particularly in the high-frequency envelopes and broadband signal, are the most prominent differences between meditation and rest. Indeed, examining the feature importances obtained from a multi-feature Random Forest classifier identified the LRTC computed from the broadband signal and the gamma1 and gamma2 signal envelopes (mainly in the regions belonging to the Frontoparietal, Default Mode, Dorsal, and Ventral Attention networks) to contribute most to the successful classification. The reduction in LRTC during meditation suggests diminished temporal redundancy, which is posited to improve processing efficiency (He 2011). This improved efficiency, which may lead to a more vivid conscious experience of the object of focus, was observed when examining the gamma envelope, which plays a crucial role in attentive processing (Jensen et al. 2007) and conscious perception (Meador et al. 2002). While Irrmischer et al. (2018a) interpreted the reduction in LRTC as a shift toward a subcritical regime, here we proposed a more nuanced interpretation based on its relationship with complexity. Specifically, we observed that decreases in LRTC co-occurred with increases in complexity measures (LZC, HFD, SpecEn), indicating that meditative states might involve a shift toward a dynamic regime where activity is less constrained by past states over long periods, allowing for a richer, more diverse, and less predictable repertoire of brain activity, which is then captured as increased complexity. This interpretation is supported by Herzog et al. (2024), who showed that weaker LRTC, coupled with flatter aperiodic slopes, may reflect diminished signal memory and increased network excitability, promoting adaptability and responsiveness during cognitively demanding or altered states. Notably, the authors argue that in this more flexible neural state, the strength of LRTC did not significantly affect task accuracy, implying that greater intrinsic network flexibility reduces the relevance of long-range dependencies. Within the framework of criticality, recent work by Thölke et al. (2025) reported a similar pattern in response to caffeine intake. The authors found increased complexity alongside flatter aperiodic slopes and reduced LRTC. These results were interpreted as a shift in brain dynamics toward increased neural excitation and closer proximity to a critical regime. We acknowledge that further computational modeling is needed to better understand how LRTC relate to excitation–inhibition balance and to the brain’s critical regime, where E:I dynamics are thought to play a key role in tuning neural activity toward or away from criticality.

Interestingly, we also found that the number of years of meditation expertise was positively correlated with the meditation-induced change in 1/f slope and gamma LRTC. Furthermore, more experienced meditators show smaller difference between rest and meditation, possibly reflecting a trait-like effect, whereby meditative brain states become more similar to resting state dynamics with continuing practice.

To further explore changes in criticality, we analyzed neuronal avalanches, which at the critical point should exhibit scale invariance and obey the crackling noise relation. Only two studies have previously explored neuronal avalanches in the context of meditation, with differing results and interpretations (Dürschmid et al. 2020; Walter and Hinterberger 2022). Neither of these studies examined the DCC, which is thought to be a more reliable indicator of criticality than solely power laws and their critical exponent values (Touboul and Destexhe 2017; Ma et al. 2019). Our finding of a decrease in DCC from both resting state and Samatha to Vipassana in experienced meditators suggests that Vipassana meditation involves a shift of brain dynamics toward the critical point. No such shift was observed from rest to Samatha meditation. Interestingly, the distinct cognitive engagements of the two meditation practices may help explain their different relationships with critical dynamics. Vipassana, as an OM-type meditation style, involves an open, unfocused attention to the body and of one’s surroundings, reminiscent of the high sensitivity to perturbations of the critical state. Meanwhile, Samatha meditation requires a focused attention on a specific object, such as the breath or a bodily sensation, aiming to achieve states of deep absorption emphasizing concentration and mental stability, which may reflect a modulation of critical brain dynamics, shifting the system toward a distinct, adaptive deviation from the critical state. Our findings thus highlight a dissociation between different meditation styles that aligns the phenomenology of these meditation states with the computational properties of the corresponding dynamical regimes.

The overall increase of the complexity index, Higuchi fractal dimension, and spectral entropy in both Samatha and Vipassana is in line with recent literature. For meditation-naive subjects performing FA meditation, Lu and Rodriguez-Larios (2022) report an increase in LZC, sample entropy (SE), and HFD compared to mind wandering. Although differences in HFD were widespread across electrodes, effects in LZC and SE were more pronounced in central electrodes. In experienced meditators, Walter and Hinterberger (2022) found higher neuronal complexity during emptiness and focused attention as compared to resting with eyes closed, as captured by the SE and HFD values. Kakumanu et al. (2018) analyzed EEG data of meditators with different levels of expertise in Vipassana practice and found increased HFD and permutation entropy in teachers and novices. An increased fractal dimension, as determined by Sevcik’s method, was also found in a calming meditation task by Vyšata et al. (2014). Only the study by Young et al. (2021) found a decrease in LZC in highly experienced meditators performing a different style of meditation practices as compared to mind wandering. The inconsistency of the results could be related to differences in meditation practices and their use of a 16-channel EEG system.

The increase in complexity during meditation observed here in source-space MEG (and other studies using scalp EEG) may be related to a larger repertoire of subjective experiences during meditation. The entropic brain hypothesis (Carhart-Harris et al. 2014; Carhart-Harris and Friston 2019) explores the connection between entropy and consciousness, suggesting that a rich altered state of consciousness, such as those induced by psychedelics, may result from an enrichment effect of neuronal dynamics, reflected by increased entropy in brain data (Atad et al. 2025). According to this theory, the degree of entropy in spontaneous brain activity captures the *richness* of conscious experience; e.g. after consuming psychedelics, EEG signals and subjective experiences become more complex and disorganized (Timmermann et al. 2019). Our findings indicate that the meditative states of Samatha and Vipassana, much like the altered states induced by psychedelics (Atad et al. 2025), generate greater neural complexity than is observed in the resting state. This similarity points to a potentially shared mechanism in how these different practices influence brain activity.

Since the relationships between these features is not always clear, we explored their relations through a feature–pair correlation analysis, following recent approaches (Walter and Hinterberger 2022; Donoghue et al. 2024; Maschke et al. 2024). Both meditation practices show a strong negative correlation between the aperiodic slope and complexity measures (LZC, HFD, SpecEn), revealing a fundamental relationship between broadband spectral characteristics and neural complexity. The study by Donoghue et al. (2024) on simulated and real data demonstrates this strong negative correlation, indicating that these measures capture shared variance in neural activity. Overall, this consistency across meditation types reinforces the relationship between a flatter aperiodic slopes and richer, more complex neural activity. However, the DCC correlations starkly differentiate the two meditation practices. Indeed, Samatha meditation is characterized by a strong positive correlation between DCC and complexity measures, and a negative correlation with the aperiodic slope. This pattern is in contrast to observations in pathological states, where increased DCC correlates with decreased complexity and steeper slope (Maschke et al. 2024). Overall, these findings warrant caution in interpretation and could suggest that Samatha may shift brain dynamics toward a different kind of criticality optimized for deep concentration and mental stability. In contrast, Vipassana meditation exhibits weaker or absent correlations between DCC and both complexity and aperiodic slope, suggesting a different engagement with critical dynamics. Vipassana appears to guide brain dynamics toward a regime characterized by a consistent decrease in DCC and a coherent alignment of other complexity measures and aperiodic slope, suggesting a type of critical dynamics that is different from Samatha. These findings offer a promising but preliminary step toward understanding how distinct meditation practices modulate brain dynamics, suggesting the existence of multiple adaptive modes within the brain’s critical regime, perhaps due to distinct modulations of E:I balance. This interpretation warrants caution and further investigation, including the exploration of additional facets of criticality, such as “edge of chaos” (Maschke et al. 2024) and chaoticity measures, and their relation to meditation could provide further insights.

Our findings complement and extend those of D’Andrea et al. (2024) who investigated the same dataset through the lens of criticality by using a microstate approach at ROI level. In their study, long-range temporal correlations and Lempel–Ziv complexity were computed on sequences of MEG microstates, providing a characterization of brain dynamics in terms of large-scale state transitions. They reported that the Hurst exponent values were reduced with respect to the RS condition, while the LZC values increased from rest to Samatha to Vipassana. However, as microstates analysis relies on clustering topographic configuration at peaks of global field power, it may overlook some important aspects of neural dynamics that may be better captured by alternative approaches (Lin et al. 2025). In this context, our analysis provides a complementary view by focusing on source-localized time series at the voxel level. We applied a broader set of complexity and criticality-related measures and introduced a direct measure of avalanche criticality, offering a more granular, region-specific characterization of meditation-induced changes in brain dynamics.

The present study has a few limitations, which can hopefully be overcome in future experiments. First, our work is limited by the relatively small sample size, which is due to the rarity of participants with such extensive meditation expertise. The correlation analyses hinting at possible trait-like effects should be viewed as exploratory, as none of the observed relationships remained statistically significant after correction for multiple comparisons. Additionally, the potential confounding effect of age on the correlation between features and expertise warrants further investigation. Furthermore, despite its widespread application, the Lempel–Ziv complexity metric used here has a number of limitations which may be overcome by exploring alternative measures (Mediano et al. 2023). Lastly, the lack of a control group limits our ability to explore trait effects among experienced meditators with greater specificity.

## Conclusions

In summary, our research reveals that both forms of meditation studied lead to significant changes in brain dynamics. These include a leveling of the 1/f slope; a consistent reduction in LRTC across gamma1, gamma2, and the broadband signal; and increases in Lempel–Ziv complexity and spectral entropy. These changes hint at an increased excitation–inhibition ratio during meditation. Importantly, DCC analysis revealed a trending differentiation between Samatha and Vipassana, with Vipassana appearing to move closer to the critical point. While previous studies often reported increases in gamma power, we observed a decrease, which may be partly explained by the correction of the power spectra for the 1/f slope. Exploratory analyses indicate that more experienced meditators may exhibit smaller changes in 1/f slope and gamma LRTC during meditation, as well as reduced DCC differences between Vipassana and Samatha which could reflect trait-like changes and convergence of brain dynamics across styles. Using a Random Forest classifier, our analysis pinpointed the LRTC for high-frequency and broadband signal as a key factor in distinguishing between meditation and resting states. This investigation into the complexity and critical dynamics of the brain during meditation opens new paths for understanding the neuronal mechanisms at play in these states, suggesting that such measurements could be crucial for deciphering how meditation affects the brain.

## Supplementary Material

Pascarella_et_al_MEG_Meditation-NCONSC_SI_niaf047-new

## Data Availability

The data analyzed in this study are subject to the following licenses/restrictions: data used in this study are protected as they might reveal confidential information about the participants. Nevertheless, data can be made available by the corresponding author upon reasonable request.
